# Alteration of GLP-1/GPR43 expression and gastrointestinal motility in dysbiotic mice treated with vancomycin

**DOI:** 10.1038/s41598-019-40978-9

**Published:** 2019-03-13

**Authors:** Xin Xu, Hirokazu Fukui, Ying Ran, Toshihiko Tomita, Tadayuki Oshima, Jiro Watari, Hiroto Miwa

**Affiliations:** 1 Division of Gastroenterology, Department of Internal Medicine, Hyogo College of Medicine, Nishinomiya, Japan; 2 Department of Gastroenterology and Hepatology, Tianjin Medical University General Hospital, Tianjin, China

## Abstract

Gut microbiota plays a pivotal role in various aspects of host physiology, including metabolism, gastrointestinal (GI) motility and hormonal secretion. In the present study, we investigated the effect of antibiotic-associated dysbiosis on metabolism and GI motility in relation to colonic expression of glucagon-like peptide-1 (GLP-1) and G protein coupled receptor (GPR)43. Specific pathogen-free (SPF) mice (ICR, 6 weeks old, female) were orally administered vancomycin (0.2 mg/ml) in drinking water for 7 days. In another experiment, germ-free (GF) mice (ICR, 6 weeks old, female) were subjected to oral fecal transplantation (FT) using a fecal bacterial suspension prepared from SPF mice that had received vancomycin treatment (FT-V) or one from untreated control SPF mice (FT-C). The gastrointestinal transit time (GITT) was measured by administration of carmine red (6% w/v) solution. The expression of GLP-1 and GPR43 was examined by immunohistochemistry and realtime RT-PCR, and the plasma GLP-1 level was measured by ELISA. In vancomycin-treated SPF mice, the diversity of the gut microbiota was significantly reduced and the abundance of *Lactobacillus* was markedly increased. Significant increases in body weight, cecum weight, plasma GLP-1 level and colonic GLP-1/GPR43 expression were also noted relative to the controls. These alterations were reproducible in GF mice with FT-V. Moreover, FT-V GF mice showed a significantly increased food intake and a significantly prolonged GITT in comparison with FT-C GF mice. Vancomycin-induced dysbiosis promotes body weight gain and prolongs GITT, accompanied by an increase of colonic GLP-1/GPR43 expression.

## Introduction

Gut microbiota play a pivotal role in various aspects of host physiology including metabolism, gastrointestinal (GI) motility and hormonal secretion^1^. Symbiotic microbiota contribute to maintenance of metabolic homeostasis and GI function, whereas gut microbiota imbalance (dysbiosis) is thought to be associated with development of metabolic diseases (obesity or diabetes) and functional gastrointestinal diseases^2^. Although the mechanism by which gut microbiota affect host metabolism and GI motility is not fully understood, bacteria producing short-chain fatty acids (SCFAs) and their corresponding G protein-coupled receptors (GPRs) are crucial for mediation of signaling between the host and gut microbiota^3^.

Glucagon-like peptide 1 (GLP-1), an incretin hormone produced by endocrine cells in the intestine, plays roles in the regulation of insulin secretion, GI motility and satiety, possibly contributing to whole-body energy metabolism^4^. Regarding the association between GLP-1 and gut microbiota, GLP-1-producing endocrine cells possess GPR 43, which can interact with SCFAs from gut bacteria^5^. Moreover, we and others have shown that transplantation of symbiotic flora to germ-free (GF) mice accelerates GI motility accompanied by alteration of GLP-1 signaling^6^, suggesting that gut microbiota may affect GI motility through modulation of GLP-1 signaling. However, the mechanism by which GLP-1 production/secretion is stimulated in the GI tract under dysbiotic condition has not been understood. Furthermore, although GPR43 is possible receptor for SCFA from gut microbiota^4,5^, it is unclear how dysbiosis affects the expression of GPR43 in the GI tract. In the present study, we prepared a dysbiotic mice by the treatment with the antibiotic vancomycin because this animal model has been widely used and its related data is well accumulated^7,8^. Thereafter, we examined how dysbiosis affects GPR43/GLP-1 expression, metabolism and GI motility.

## Materials and Methods

### Animals

Specific pathogen-free (SPF) mice (ICR, 6 weeks old, female) and germ-free (GF) mice (ICR, 6 weeks old, female) were obtained from Clea Japan (Tokyo, Japan) and used for the following experiments. The experimental protocol was approved by the Animal Use and Care Committee at Hyogo College of Medicine. In addition, all experiments described below were performed in accordance with relevant guidelines and regulations.

### Antibiotic treatment and fecal transplantation

To create dysbiotic conditions for gut microbiota, SPF mice were orally administered vancomycin (0.2 mg/ml; Sigma, Saint Louis, MO, USA) in drinking water for seven days, whereas controls were supplied with untreated water^9,10^.

To examine the effect of dysbiotic flora on host physiology, fecal transplantation (FT) was performed as reported previously^11,12^. The fecal suspensions were freshly prepared from SPF mice after seven days of vancomycin treatment by 10-fold dilution of colonic content with saline, and then orally administered to GF mice to reconstitute the dysbiotic intestinal flora. As controls, fecal suspensions from SPF mice that had not received vancomycin treatment were similarly administered to GF mice. After FT, the GF mice were housed under SPF conditions for five weeks.

Body weight and 24-h food intake were monitored weekly. To measure the amount of food intake for mice, the experimental mice was housed and feed in a cage separately for 24 hours, the weight of food was measure before and after. The 24 h food intake was calculated as the difference between before and after food weight. At the end point of the experiments, the mice were fasted for 4 h before sacrifice. The length of the small intestine and colon, and the weight of the cecal content, were measured. The GI tissues were removed from the mice, cut open along the longitudinal axis, rinsed with saline, and fixed in neutral aqueous phosphate-buffered 10% formalin for histological examination or stored in nitrogen liquid for real-time RT-PCR.

### Real-time RT-PCR

Total RNA was isolated from GI tissues with Trizol reagent (Invitrogen, Carlsbad, CA). Total RNA (4 ug) was reverse-transcribed using oligo-dT primer (Applied Biosystems, Branchburg, NJ), and real-time RT-PCR was performed using 7900 H Fast Real-Time PCR System (Applied Biosystems) as previously described^13^. The set of primers for mouse *proglucagon, GPR43, and GAPDH* were prepared as shown in Table 1. Real-time RT-PCR assays were carried out with 200 ng of RNA equivalent cDNA, SYBR Green Master Mix (Applied Biosystems), and 500 nmol/l gene-specific primers. The PCR cycling conditions were 50 °C for 15 s and 60 °C for 60 s. The intensity of the fluorescent dye was determined, and the expression levels of target gene mRNA were normalized to *GAPDH* mRNA expression levels.

**Table 1:** Primers for real-time RT-PCR analysis.

*Proglucagon*	Forward	5′-TGAGATGAGCACCATTCTGGA-3′
	Reverse	5′-TCCGCAGAGATGTTGTGAAGA-3′
*GPR43*	Forward	5′-ACAGTGGAGGGGACCAAGAT-3′
	Reverse	5′-GGGGACTCTCTACTCGGTGA-3′
*GAPDH*	Forward	5′-GGAGAAACCTGCCAAGTATG-3′
	Reverse	5′-TGGGAGTTGCTGTTGAAGTC-3′

### Immunohistochemistry

Immunohistochemical staining for GLP-1 and GPR43 was performed with an Envision Kit (Dako, Kyoto, Japan) according to the manufacturer’s protocol, using anti-GLP-1 antibody (dilution 1:1000; Abcam, Cambridge, UK), anti-GPR43 antibody (dilution 1:50; MyBioSource, Diego, USA). In brief, the sections were deparaffinized, rehydrated, and treated by microwave heating for 20 min in 1 Dako REAL Target Retrieval Solution (Dako Denmark, Glostrup, Denmark) as previously described^14^. To quench endogenous peroxidase activity, the sections were preincubated with 0.3% H_2_O_2_ in methanol for 20 min at room temperature. The sections were then incubated with primary antibodies for 60 min at room temperature. Thereafter, the slides were washed in PBS, incubated with horseradish peroxidase-conjugated secondary antibody for 30 min, visualized by 3,3′-diaminobenzidine tetrahydrochloride with 0.05% H_2_O_2_ for 3 min, and then counterstained with Mayer’s hematoxylin. The number of GLP-1-positive and GPR43-positive epithelial cells were evaluated as follows: Five sections in each mouse were prepared for the small intestine and colon, respectively. The positive cells were counted in at least five different visual fields in a 1,000-μm stretch of the entire length with well-oriented tissue sections, and the average was calculated in each mouse.

### ELISA assay

Blood samples were collected into 1.5-ml tubes containing 2 mg EDTA-2Na (Wako, Osaka, Japan) and 15 μl dipeptidyl peptidase IV inhibitor (Merck, NJ, USA), an enzyme that degrades active GLP-1 into its inactive form. Blood samples were centrifuged at 1300 × g for 10 min at 4 °C to isolate the plasma. ELISA assay kits for active GLP-1 were obtained from IBL (Gunma, Japan) and utilized according to the manufacturer’s instructions to determine active GLP-1 levels using a SpectraMax Plus 384 Microplate Reader (Molecular Devices, California, USA).

### Gastrointestinal transient time

GI transient time (GITT) was measured as previously described^11,15^. In brief, the mice received orally 0.3 mL of 0.5% methylcellulose solution including 6% carmine red (Wako, Osaka, Japan). After administration of the solution, mice were left free for food and water ad libitum until the first red fecal pellet appeared. GITT was determined as the time period between the gavage and the appearance of the first red fecal pellet^16^.

### Extraction of DNA from fecal samples

Extraction of bacterial DNA was performed as described previously^17^. In brief, the fresh fecal samples were resuspended in a solution containing 450 μl of extraction buffer (100 mM Tris-HCl, 40 mM EDTA; pH 9.0) and 50 μl of 10% sodium dodecyl sulfate. Then, 300 μg of glass beads (diameter, 0.1 mm) and 500 μl of buffer-saturated phenol were added to the suspension, and 400 μl of the supernatant was collected. The DNA was eluted from the supernatant by phenol-chloroform method.

### Illumina library generation and DNA sequencing

Analysis of the 16S rDNA of the microbial community present in feces was performed in accordance with a method described previously^18^ with minor modifications. In brief, the V3-V4 region of 16S rDNA was amplified using the primers as previously reported^18^, and then ligated with overhang Illumina adapter consensus sequences. After PCR reactions,the amplicon was purified using AMPure XP magnetic beads (Beckman Coulter, Brea CA, USA). The Illumina Nextera XT Index kit (Illumina) with dual 8-base indices was used to allow for multiplexing. To incorporate two unique indices to the 16S amplicons, PCR reactions were performed as previously described^19^. The libraries were purified by AMPure XP beads, quantified fluorometrically using a QuantiT PicoGreen ds DNA Assay Kit (Invitrogen, Paisley, UK) and then diluted to 4 nM using 10 mM Tris-HCl (pH 8.0), followed by pooling of the same volume for multiplex sequencing. The multiplexed library pool (10 pM) was spiked with 40% PhiX control DNA (10 pM) to improve base calling during sequencing. Sequencing was conducted using a 2 × 250-bp paired-end run on a MiSeq platform with MiSeq Reagent Kit v2 chemistry (Illumina).

### DNA sequence analysis

Demultiplexing and removal of indices were performed using the MiSeq Reporter software (Illumina) as previously reported^19^. Filtering out of low-quality sequences, removal of chimera sequences, construction of operational taxonomic units (OTUs), and taxonomy assignment were conducted using the Quantitative Insights Into Microbial Ecology (QIIME) pipeline (http://qiime.org/)^20^. In brief, 30000 raw reads were randomly obtained from the sequence files for each sample and merged by fastq-join with the default setting. The sequence reads with an average quality value of <25 were removed, and then chimera-checked. Five thousand high-quality sequence reads were randomly obtained for each sample, and OTUs for total high-quality reads were constructed by clustering with a 97% identity threshold. The representative reads of each OTU were then assigned to the 16S rRNA gene database by using UCLUST with ≥97% identity. Each taxon in gut microbiota was compared at genus level. The Shannon index was calculated to investigate the alpha diversity of microbiota in the samples.

### Statistical analysis

All statistical analyses were conducted with the R statistical software version 3.1.3^21^. Data are expressed as means ± SE. Significance of differences between two animal groups was analyzed by Mann-Whitney *U*-test. Difference were considered to be significant at *P* < 0.05. In the analyses of gut microbiota, statistical significance was determined by Welch’s t test with Benjamini-Hochberg correlation.

### Ethics approval and consent to participate

The animal experiments were carried out with the approval of the Animal Use and Care Committee at Hyogo College of Medicine.

## Results

### Effect of vancomycin treatment on the structure of gut microbiota in mice

**Figure 1:**
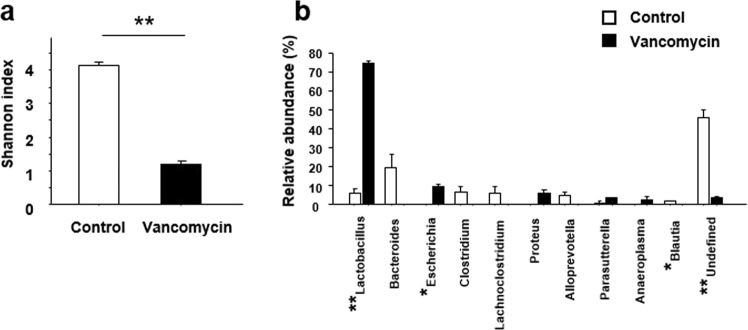
Effect of treatment with vancomycin for seven days on gut microbiota. (**a**) Alpha-diversity of the gut microbiota. Shannon index calculated from the observed OTU numbers of intestinal microbiota samples from control and vancomycin-treated mice. (**b**) Relative abundance of intestinal bacteria. The relative abundance of each bacterial genus was analyzed by next-generation sequencing of bacterial 16S rDNA. The results are presented as the mean ± SE (n = 3 in each group). Significant differences between the control and vancomycin-treated groups at *P < 0.01 and **P < 0.001. Statistical significance was determined by Welch’s t test with Benjamini-Hochberg correction.

To confirm whether vancomycin treatment caused dysbiosis in the experimental mice, we analyzed gut microbiota profile. The alpha-diversity of the gut microbiota was significantly lower in vancomycin-treated mice than in the controls (Fig. 1a). Moreover, we examined the genera of gut microbiota present in the experimental mice. Among 10 major genera, *Lactobacillus* was markedly increased in the vancomycin-treated mice (Fig. 1b). In addition, *Escherichia* was significantly increased, whereas *Blautia* was less abundant in vancomycin-treated mice than in the controls (Fig. 1b).

### Effect of vancomycin treatment on body weight and intestinal morphology in mice

**Figure 2:**
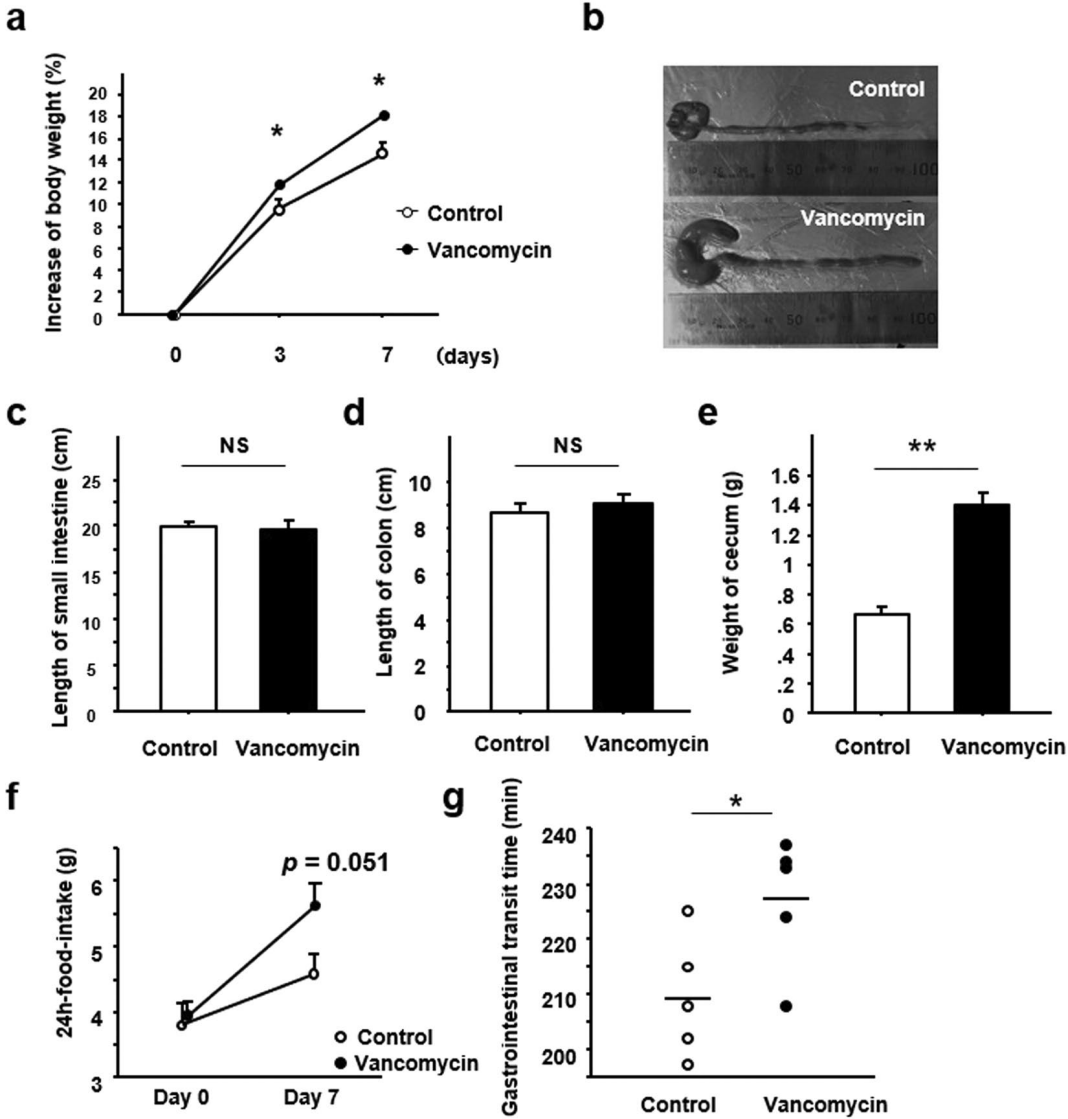
Effect of vancomycin treatment for seven days on body weight, food-intake, Gastrointestinal transit time and intestinal morphology. (**a**) Change in body weight. (**b**) Representative appearance of the cecum in the experimental mice treated with vancomycin. Length of (**c**) small intestine and (**d**) colon. (**e**) Weight of cecum. (**f**) Change in food-intake. (**g**) Gastrointestinal transit time. Results are expressed as the mean ± SE (n = 5 in each group). Significantly greater than control at the same time point: *P < 0.05, **P < 0.01. NS, not significant.

Body weight increased according to body growth in both the control and vancomycin-treated groups. The percentage increase in body weight was significantly greater in vancomycin-treated mice from 3 days after the start of the experiment (Fig. 2a). Observation of intestinal morphology demonstrated that the cecum was apparently enlarged in the vancomycin-treated mice relative to the controls (Fig. 2b). Although the lengths of the small intestine and colon did not differ between the two groups, cecum weight was significantly greater in the vancomycin-treated mice (Fig. 2c–e). The amount of food-intake was greater in the vancomycin-treated mice compared with control (Fig. 2f). GITT was significantly prolonged in the vancomycin-treated mice relative to control (Fig. 2g).

### Effect of vancomycin treatment on expression of GLP-1 and GPR43 in the colon of mice

**Figure 3:**
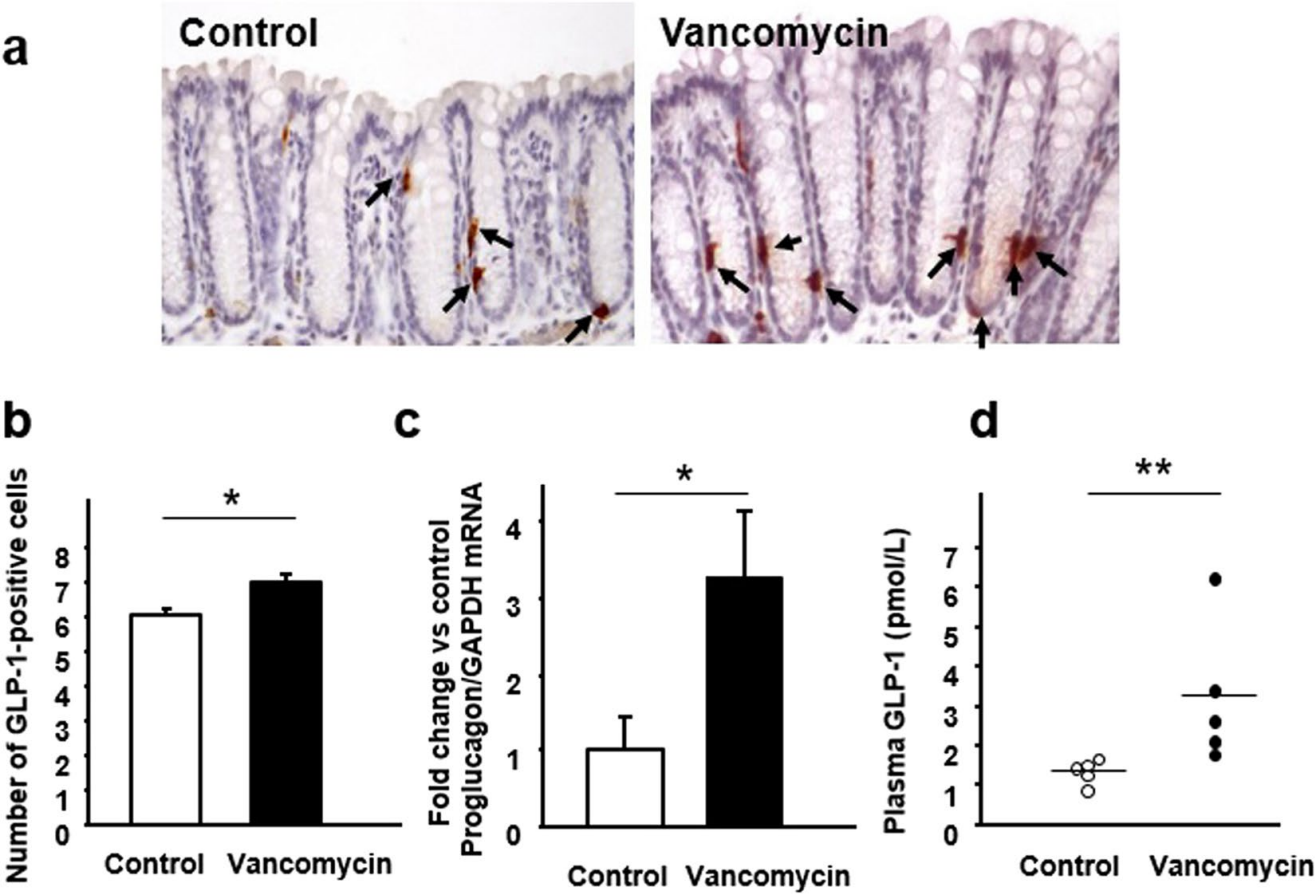
Effect of vancomycin treatment for seven days on colonic GLP-1 expression and the plasma GLP-1 level. (**a**) Immunostaining for GLP-1 in the colonic mucosa. (**b**) The number of GLP-1-positive cells in the colonic mucosa. (**c**) Expression of *proglucagon* mRNA in colon tissues. (**d**) Plasma GLP-1 level. Results are expressed as the mean ± SE (n = 5 in each group). Significantly greater than the control: *P < 0.05.

GLP-1 was expressed in colonic epithelial cells with an ovoid or pyramidal shape (Fig. 3a). The number of GLP-1-positive cells in the colonic mucosa was significantly greater in vancomycin-treated than in untreated mice (Fig. 3a,b). Consistent with this finding, the expression of mRNA for *proglucagon* (the gene encoding GLP-1) was significantly increased in the mice that had received vancomycin (Fig. 3c), and moreover, the plasma GLP-1 level was significantly elevated in those mice relative to the controls (Fig. 3d).

**Figure 4:**
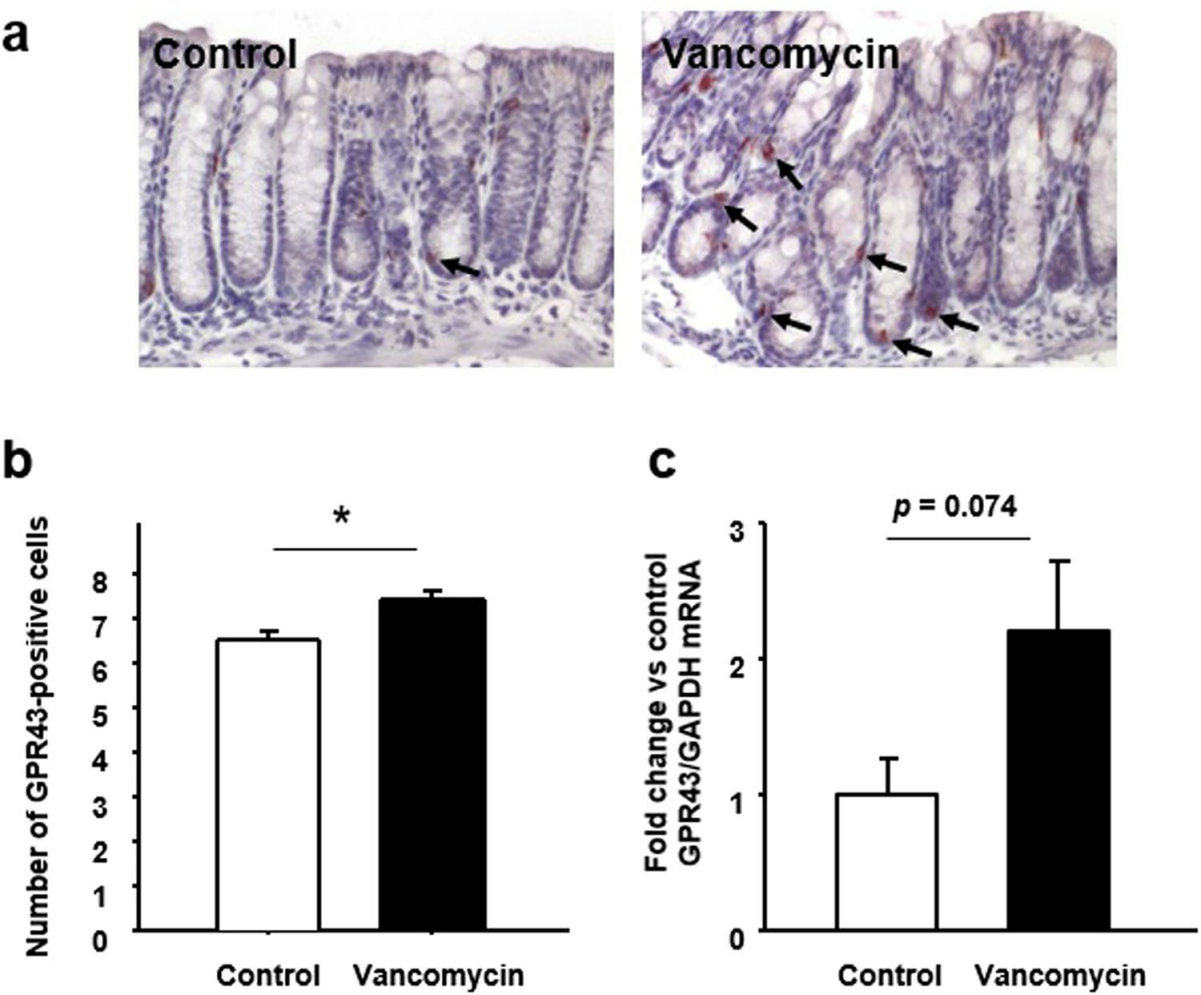
Effect of vancomycin treatment for seven days on colonic GPR43 expression. (**a**) Immunostaining for GPR43 in the colonic mucosa. Arrows indicating positive cells. (**b**) The number of GPR43-positive cells in the colonic mucosa. (**C**) Expression of *GPR43* mRNA in the colon tissues. Results are expressed as the mean ± SE (n = 5 in each group). Significantly greater than control: *P < 0.05.

Immunoreactivity for GPR43 was also localized in the ovoid or pyramidal epithelial cells of the colonic mucosa, the morphology being consistent with gut endocrine cells (Fig. 4a). The number of GPR43-positive cells in the colonic mucosa was significantly increased in the mice treated with vancomycin (Fig. 4a,b), and the level of expression of *GPR43* mRNA tended to be higher in those mice relative to the controls (Fig. 4c).

### Effect of vancomycin-induced gut microbiota alteration on gastrointestinal morphology and physiology

**Figure 5:**
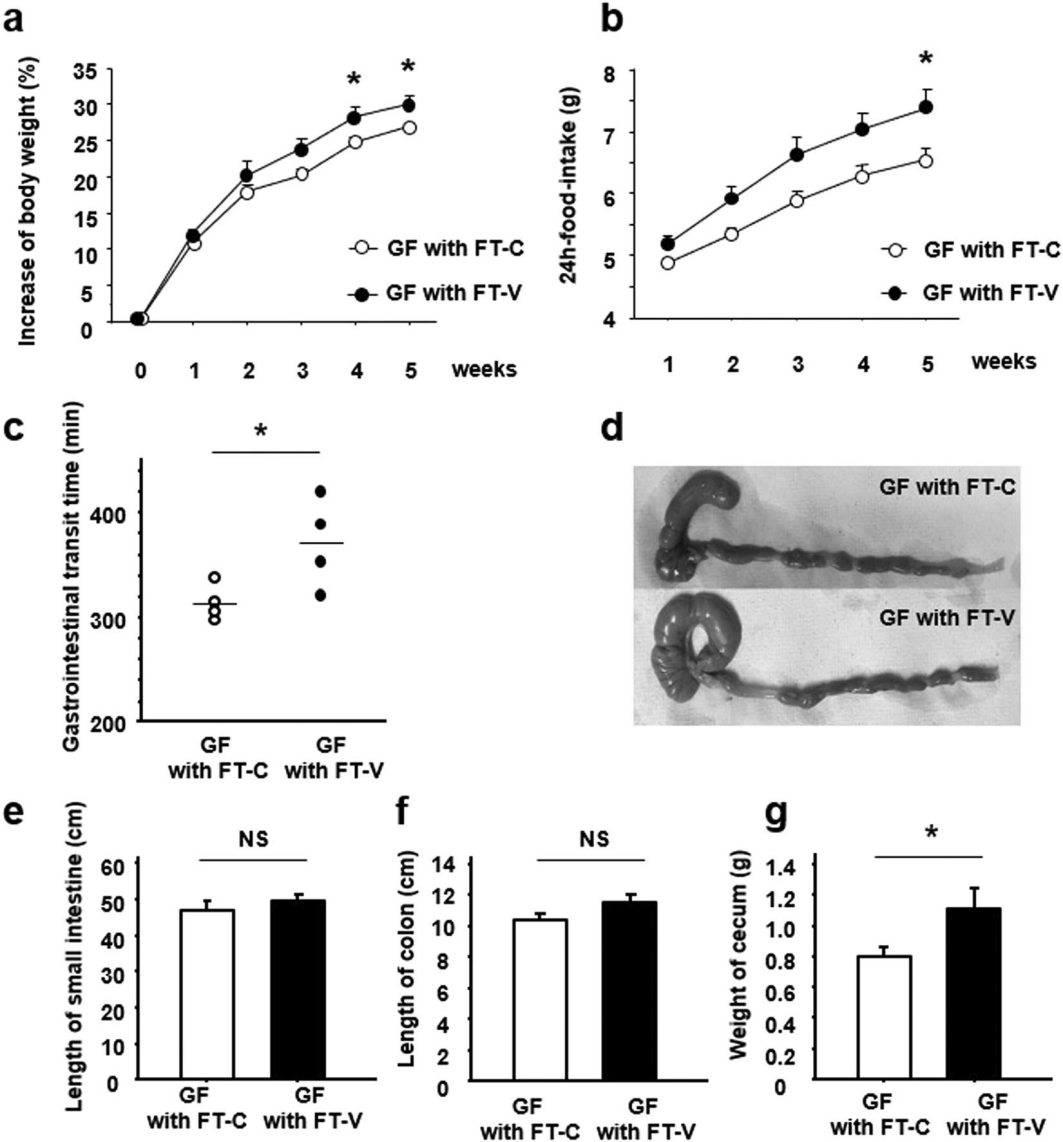
Effect of vancomycin-induced gut microbiota alteration on gastrointestinal morphology and physiology in germ-free (GF) mice. Change in (**a**) body weight and (**b**) food-intake in GF mice subjected to fecal transplantation (FT) using samples from vancomycin-treated mice (FT-V) or from control mice (FT-C). (**c**) Gastrointestinal transit time in GF mice with FT-V or FT-C. (**d**) Representative appearance of the cecum in GF mice with FT-V or FT-C. Length of (**e**) small intestine and (**f**) colon. (**g**) Weight of cecum. Results are expressed as the mean ± SE (n = 4 in each group). Significantly greater than GF mice with FT-C at the same time point: *P < 0.05. NS, not significant.

To examine whether the characteristic features evident in vancomycin-treated mice were due to alterations of gut microbiota, we transplanted the gut flora from those mice into GF mice. From 2 weeks after the start of the experiment, GF mice that had undergone FT using samples from vancomycin-treated mice (FT-V) showed a greater gain in body weight. At 5 weeks after FT, the gain of body weight was significantly greater in GF mice with FT-V than in those that had undergone FT using samples from control mice (FT-C) (Fig. 5a). We then studied the changes in food intake in the two groups. Similarly to body weight, food intake became greater in GF mice with FT-V from 2 weeks after the start of the experiment, and these mice subsequently showed a significant increase at 5 weeks (Fig. 5b). Moreover, we found that the GITT was significantly prolonged in the GF mice with FT-V relative to GF mice with FT-C (Fig. 5c).

Histological investigation demonstrated enlargement of the cecum in GF mice with FT-V, being similar to that in the vancomycin-treated mice (Fig. 5d). The lengths of the small intestine and colon did not differ between GF mice with FT-V and GF mice with FT-C (Fig. 5e,f), but cecum weight was significantly greater in the former (Fig. 5g), being compatible with the relationship between the vancomycin-treated mice and the controls.

### Effect of gut vancomycin-induced microbiota alteration on expression of GLP-1 and GPR43 in the colon

**Figure 6:**
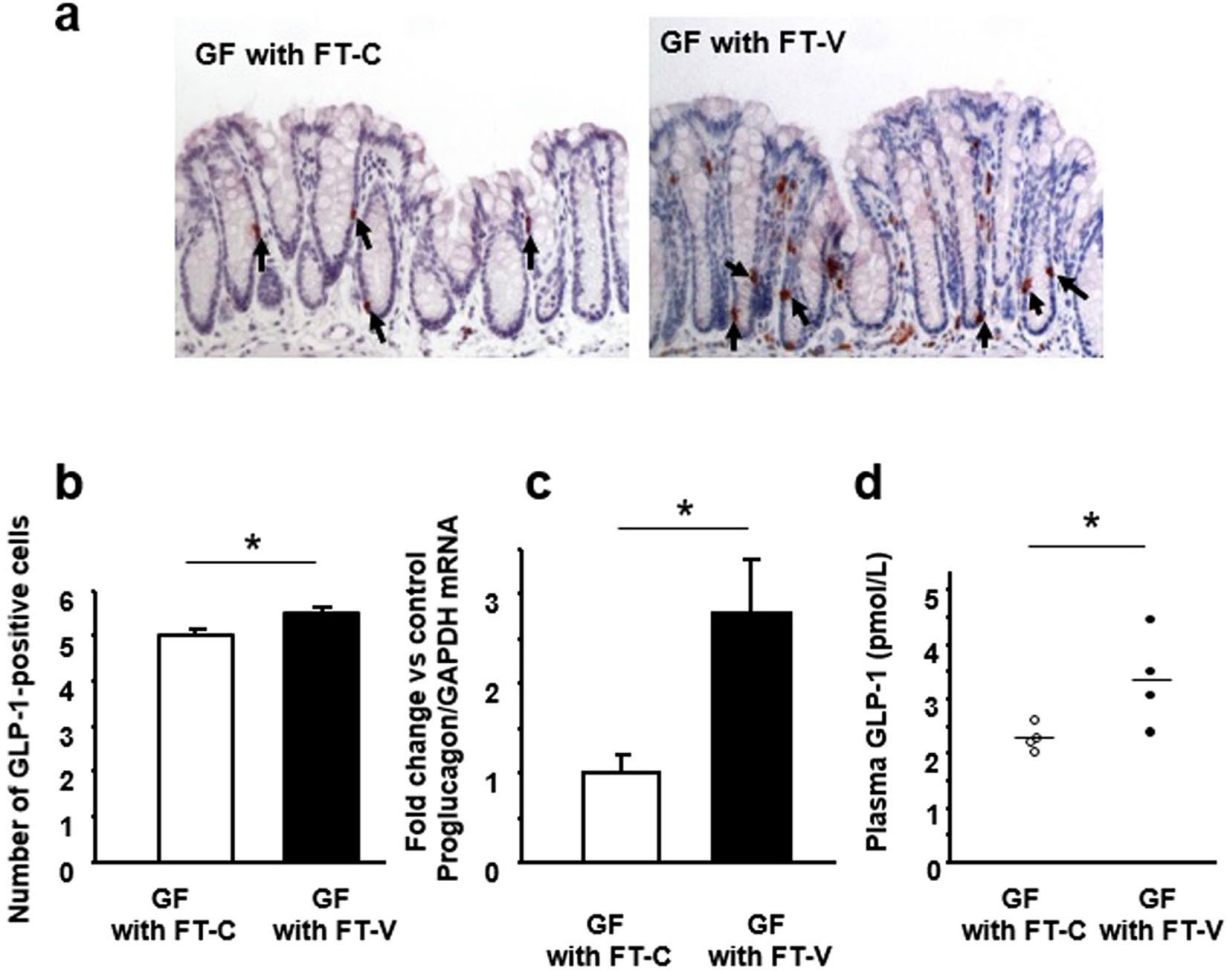
Effect of vancomycin-induced gut microbiota alteration induced on colonic GLP-1 expression and the plasma GLP-1 level in germ-free (GF) mice. (**a**) Immunostaining for GLP-1 in the colonic mucosa of GF mice subjected to fecal transplantation (FT) using material from vancomycin-treated mice (FT-V) or from control mice (FT-C). (**b**) The number of GLP-1-positive cells in the colonic mucosa. (**c**) Expression of *proglucagon* mRNA in colon tissues. (**d**) Plasma GLP-1 level. Results are expressed as the mean ± SE (n = 4 in each group). Significantly greater than GF mice with FT-C: *P < 0.05.

The number of GLP-1-positive cells in the colonic mucosa was significantly higher in GF mice with FT-V than in GF mice with FT-C (Fig. 6a,b). The expression of *proglucagon* mRNA in the colon was also significantly increased in GF mice with FT-V (Fig. 6c), and in fact the plasma GLP-1 level was significantly elevated in those mice relative to GF mice with FT-C (Fig. 6d).

**Figure 7:**
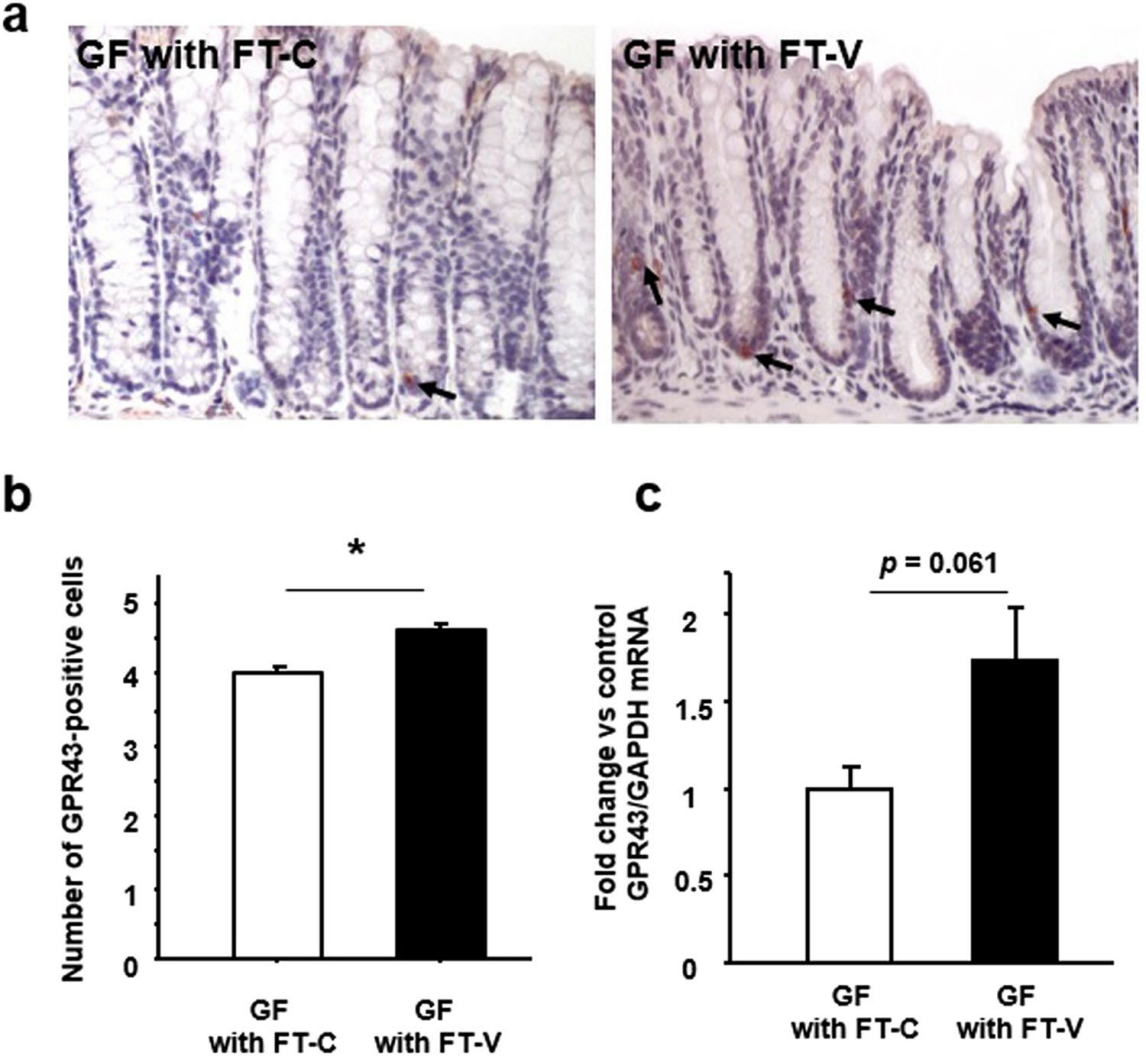
Effect of vancomycin-induced gut microbiota alteration on colonic GPR43 expression in germ-free (GF) mice. (**a**) Immunostaining for GPR43 in the colonic mucosa of GF mice subjected to fecal transplantation (FT) using material from vancomycin-treated mice (FT-V) or from control mice (FT-C). Arrows indicating positive cells. (**b**) The number of GPR43-positive cells in the colonic mucosa. (**c**) Expression of *GPR43* mRNA in colon tissues. Results are expressed as the mean ± SE (n = 4 in each group). Significantly greater than GF mice with FT-C: *P < 0.05.

We also investigated the expression of GPR43 in the colonic mucosa of GF mice with FT. GPR43 immunoreactivity was observed in epithelial cells such as endocrine cells, and the number of immunoreactive cells was significantly higher in GF mice with FT-V than in those with FT-C (Fig. 7a,b). Although the difference was not statistically significant, the level of expression of *GPR43* mRNA tended to be higher in GF mice with FT-V than in those with FT-C (Fig. 7c).

## Discussion

It has recently been reported that commensal gut microbiota are involved in the regulation of GLP-1^6,11^, which plays a pivotal role in not only insulin-associated energy metabolism but also GI motility^22^. In the present study, we investigated the effect of dysbiosis on GLP-1 expression and found that the expression of GLP-1 was increased in the colon of mice that had been treated with vancomycin. Supporting our data, a few studies have reported that the number of GLP-1-positive cells and/or the plasma GLP-1 level is increased in mice after treatment with vancomycin alone or a combination of vancomycin and other antibiotics^10,23^. GLP-1 is produced by enteroendocrine L cells and its production and secretion are regulated by carbohydrates, fatty acids, amino acids and hormonal factors^24^. Therefore, such factors are likely involved in the enhancement of GLP-1 expression resulting from vancomycin treatment. In particular, since short-chain fatty acids (SCFAs) are produced mainly by gut microbiota and become the energy source for colonic epithelial cells^1^, SCFAs may play a key role in mechanism by which vancomycin-induced dysbiosis causes enhancement of GLP-1 expression in the intestinal tract^10^. In this study, we were unable to evaluate the specific SCFAs involved because of methodological limitations; however, we have clarified that the expression of GPR43, a possible receptor for SCFA in GLP-1-producing L cells, is enhanced in colonic epithelial cells. This finding suggests that GLP-1-producing cells might be sensitive to extracellular stimuli, and partly involved in the enhancement of GLP-1 expression.

Although several phenotypic characteristics, including enhancement of GLP-1/GPR43 expression, an increase of body weight gain and enlargement of the cecum, was observed in dysbiotic mice after vancomycin treatment, it was still debatable whether those characteristics were in fact due to alteration of the gut microbiota. Therefore, we subjected GF mice to transplantation of material from vancomycin-treated mice to clarify whether the above features were reproducible. This revealed that GF mice with FT-V not only showed an increase in the basal GLP-1/GPR43 level and body weight gain but also enlargement of the cecum, supporting the contention that vancomycin-induced dysbiosis was related to those characteristics. Examination of the gut microbiota profile revealed a marked increase of *Lactobacillus* in mice after vancomycin treatment, being compatible with the findings of a few recent studies^8,9,25^. Interestingly, it has been reported that administration of probiotic *Lactobacillus* strains promotes not only SCFA production^26^ but also GLP-1 secretion^27,28^. Together, these findings suggest that the increase of GLP-1 expression in vancomycin-treated mice is linked to the marked increase of gut *Lactobacillus* strains in those mice.

What is the role of enhanced GLP-1 expression in mice with vancomycin-induced dysbiosis? GLP-1 plays a role in not only energy metabolism but also GI motility, and therefore we investigated the effect of vancomycin-induced dysbiosis on the GLP-1/GI motility axis. This revealed that the GF mice with FT-V had a suppressed GI motility accompanied by up-regulation of GLP-1. It still remains unclear whether these alterations of GI motility and gut hormone balance are functional disorders resulting from gut dysbiosis or simply a reaction to dysbiosis-associated pathophysiology. Although we are unable to address this significant issue, the metabolic disorders such as increased body weight and food intake in GF mice with FT-V are of interest. It is known that antibiotic treatment, especially in early life, alters the structure of the gut flora and is frequently linked to the development of obesity^29^. Indeed, vancomycin treatment appears to lead to an increase of body weight and/or body fat in mice^25,29^, consistent with our data. In the present study, we found that *Lactobacillus* is increased in the vancomycin-treated mice whose body growth is promoted. In this context, it is interesting that *Lactobacillus* is increased in obese with insulin resistance^30,31^ and moreover, *Lactobacillus* species are widely used as growth promoters in the farm industry^32^. On the other hand, it has been known that the increase of GLP-1 is likely found in obese patients with insulin resistance^33^. Although we have no exact answer for the discrepancy between the promotion of food intake/body weight gain and the increase of appetite suppressive GLP-1, it is tempting to speculate that the up-regulation of GLP-1 may be a protective reaction against the dysbiosis-associated glucose and/or lipid metabolism dysfunction. If the increase in expression of GLP-1 is a reactive response to obesity, GLP-1-associated suppression of GI motility would be useful to discourage food intake. On the other hand, it is still unclear whether the amount of SCFA, which acts as an energy source for colonic epithelial cells, is increased or decreased in vancomycin-treated mice^6,29^. From the view point of energy harvest in the colonic lumen, GLP-1-associated suppression of GI motility may be helpful to reduce intake of any source material for bacterial fermentation when the amount of SCFA is increased in the colon^1^. In contrast, when the amount of colonic SCFA is decreased, suppression of GI motility may advantageous for absorption of SCFA by colonic epithelial cells^6^. In this context, it seems very difficult to interpret the significance of the altered GLP-1/GI motility axis in GF mice with FT-V.

In summary, we have shown that treatment of mice with the antibiotic vancomycin causes dysbiosis of gut microbiota, and increases the expression of GLP-1 and GPR43 in the colonic mucosa. Moreover, we have demonstrated that the enhancement of GLP-1 and GPR43 expression is reproducible in GF mice with FT-V, accompanied by an increase of body weight gain and prolongation of the GITT. These findings confirm that vancomycin-induced dysbiosis is responsible for the increase of GLP-1 expression and development of an obese phenotype, although it is still unclear whether the alteration of GI motility represents a protective reaction against dysbiosis-associated pathophysiology. In this context, in further studies, we will need to investigate the metabolites present in the GI tract and/or the effect of probiotics on vancomycin-associated dysbiosis and its related pathophysiology.

## Data Availability

All data generated or analyzed during this study are included in this published article.
